# A Danger to Be Averted

**Published:** 1892-11-05

**Authors:** 


### The Hospital

An Institutional Journal of

**Science, Medicine, Nursing, and Philanthropy.**

*For Hospitals, Asylums, Infirmaries, Dispensaries, Medical Practitioners, Students, Nurses, and the Charitable Public.*

Vol. XIII.—No. 319.] [Nov. 5, 1892.

### A Danger to be Averted.

Competent physicians have expressed the opinion that these islands will probably be visited by cholera in the coming spring. Death-bed repentances are proverbially of little avail in affairs spiritual; they are of no avail at all in bodily concerns. We therefore invite all thinking readers to consider with us one great source of danger which looms in the immediate future, and which may, by reasonable measures, promptly taken, be reduced to a minimum.

A deputation of the unemployed waited upon the Rev. Dr. Parker at the City Temple, after the mid-day service, last Thursday. The conference between the deputationists and the preacher of the City Temple had its comical aspect. Dr. Parker listened to what the deputation had to say, gave them a little sermon of the wet blanket kind from the text *non possumus*, and finally dismissed each member of the little company with a gratuity of half-a-crown. This method of dealing with the question had at least the merit of originality.

The significance of this odd demonstration lay in the fact which one member stated—that there are at the present time more than two hundred thousand people in East London who are in a state of semi-destitution, some of them being on the verge of positive starvation. These people consist of men and women who have no employment and cannot find any, and of the children and aged people who are dependant upon them. Our object just now is not to excite pity for all those half-naked and hungry men, women, and children, but to consider them, in a strictly scientific spirit, as seed-beds in the process of preparation for the broadcast of cholera, which we are told is to be sown among them in the coming spring. Those two hundred thousand, if they are in the first stage of destitution at the beginning of November, will be in such a condition of sickness and wretchedness before the spring comes round that the very mildest inflow of cholera will sweep them away by thousands and tens of thousands.

There are those who would say that such a consummation would be a great advantage to the rest of the community. We do not believe this; and even those who do believe it must remember that there is a certain cost attaching to such a process. That cost they and we must consider. If it should come to pass that those two hundred thousand continue to starve until the spring, and that in the spring the cholera comes and kills half of them, it is probable that every one of the cholera-slain hundred thousand will be followed into the grim shades by at least five healthy persons who have never known what poverty and starvation were. This is the possibility which stares us full in the face. The navigator who invades an unfamiliar sea keeps a sharp look-out ahead for rocks and other dangerous possibilities. He never steers his barque upon certain destruction with his eyes open. If he sees rocks and breakers ahead he changes his course. He were a madman otherwise. Are not these starving two hundred thousand like dangerous rocks ahead in mid-ocean? And do not they appear a thousandfold more alarming when the terrible spectre of cholera is seen hovering about the rocky peaks? These are grave matters for all of us who can think, and who are conscious of responsibility.

Something must be done for the hungry two hundred thousand, and it must be done with promptitude and efficiency before they are reduced to feeble health, and to the condition of dangerous centres of infection for the spread of cholera. Something, we say, must be done! The question is—What? Have we the power to deal effectively with two hundred thousand people, out-of-works and their children, to find them with work, and to keep them in health? Let us answer this question by asking another—Have we the power to deal promptly and effectively with a foreign army of two hundred thousand men if we were invaded by such an army? The proper answer to that is—Let the army invade us, and we shall see! But whether would be easier—to deal effectively with two hundred thousand of our own loyal people, many of whom only require wise guidance and a modicum of temporary help, or to wrestle in a death-struggle with a powerful foreign foe? To deal with our own people would be a mere summer holiday compared with the quelling of a formidable foreign invasion.

The question of practical schemes remains to be considered. There is no lack of advisers. We may do our work vicariously by means of "General" Booth; or we may spend the months of winter in microscopical investigation like the Charity Organization Society—the people meanwhile kindly dying by thousands, and making the relief of the rest much easier, if any of them should perchance be found worthy of it. But is this all that we can do; or is it the best we can devise? There is another scheme in operation which, we venture to suggest, is worthy of careful consideration at the present time. That scheme is the method successfully carried out by the association known as the "North Kensington Friendly Workers." Now the way to really discover what this scheme can do is to find out what it actually has done. In sober fact it has done these two things—relieved the destitution of the destitute, and provided the workless with work.

This is a great claim to make on behalf of a young association. Is it justified by facts? By way of answer to this question, readers are referred to a pamphlet by a well-known journalist, Mrs. Furley Smith, copies of which can be obtained at the offices of the association, 28, Colville Square, W. The pamphlet gives the history and working of the association from its commencement, two years ago, to the present time. The North Kensington Friendly Workers claim to have solved the problem of the "Inner life of poorer London," at any rate so far as North Kensington is concerned.

Now, if this North Kensington scheme were the very best that has ever been planned, or indeed that ever could be planned, we cannot expect all the world to see its perfection in a moment. But there are certain points about it which strike the writer, who himself has had nothing to do either with its inception or its working, as being very specially worthy of consideration. In the first place, it is a homogenous scheme; it unites all the available workers of every name and denomination under one central guidance and authority; it routs sectarianism and overlapping at a blow. In the second place, it is thorough; it carries its operations into every street, every house, every tenement in which the working poor are congregated together. Its voluntary agents consist of a well drilled army of men and women of some leisure: and the work of the association is so well planned that every street, house, and tenement can be kept under constant supervision. In the third place, the association consists of "friendly workers," not of mere "inquisitors." It knows the normal character and daily life of those whom it serves; and thus, when help is needed, there is no time wasted in investigation, because the facts of each poor family's history are already known. The association makes this large general claim—that for the last two years it has relieved the destitution of the North Kensington district, and supplied its workless with work. Are these claims justified by facts? We believe that, broadly speaking, they are; and if they are, then the North Kensington Friendly Workers are justified in asking the Lord Mayor, the Members of Parliament, the County Councillors of London, and all sorts and conditions of practical philanthropists to consider their scheme, with a view to its immediate and general adoption. Those who see the rocks of cholera ahead in the spring which is before us, should take all these facts immediately to heart.

